# Non-suicidal Self-Injury Among Adolescents From Diverse Ethnocultural Groups in Israel: The Association With Sleep Problems and Internet Addiction

**DOI:** 10.3389/fpsyt.2022.899956

**Published:** 2022-05-13

**Authors:** Sami Hamdan, Alan Apter, Yossi Levi-Belz

**Affiliations:** ^1^School of Behavioral Sciences, The Academic College of Tel Aviv-Yaffo (MTA), Tel Aviv, Israel; ^2^Fienberg Child Study Center, Schneider Children' Medical Center of Israel, Tel Aviv University, Tel Aviv, Israel; ^3^The Lior Tsfaty Center for Suicide and Mental Pain Studies, Ruppin Academic Center, Hadera, Israel

**Keywords:** Non-suicidal Self-Injury (NSSI), sleep problems, ethnicity, internet addiction, depression

## Abstract

**Objectives:**

Although Non-suicidal Self-Injury (NSSI) has received more attention in recent years, most of these studies focused on samples from North American and European countries; consequently, little is known about its patterns and frequency in other cultures as well as its relation to sleep problems and internet addiction. As one of the few studies that aim to fill this gap, the current study examined the prevalence, characteristics, and types of NSSI behaviors among adolescents from diverse ethnocultural groups.

**Methods:**

A sample of 642 adolescents, aged 12–18 years, were randomly recruited from different middle and high schools in Israel, employing a snowball sampling technique. The sample included the following: 50% Jews and 34.7% Muslims born in Israel, 9.7% immigrants from the former Soviet Union (FSU), and 4.4% immigrants from Ethiopia. The participants completed self-report questionnaires that assessed their NSSI, sleep problems, internet addictions, and depressive symptoms.

**Results:**

Almost one-third of the sample had engaged in NSSI, while 6% frequently injured themselves. More than half of the FSU immigrants and one-third of the Muslim participants indicated that they engaged in NSSI. These two population groups also exhibited severe depressive symptoms, sleep problems, and internet addictions. The most parsimonious correlations with NSSI included being male, an immigrant/Muslim minority who exhibited severe depressive symptoms and internet addictions.

**Conclusions:**

These results emphasize the need for routine NSSI assessments to prevent long-term sequelae, including any forms of suicidal thoughts and behaviors and adult borderline personality disorder (BPD). Primary preventive programs that include adaptive coping skills may eliminate the social contagion effect of NSSI.

## Introduction

Non-suicidal Self-Injury (NSSI) refers to the deliberate and direct wounding of one's body tissue without suicidal intent (1, 2). This phenomenon affects individuals worldwide, and its occurrence has increased rapidly in the last two decades (3, 4). Most individuals who engage in NSSI tend to utilize more than one method to injure themselves repeatedly (5–8). People engage in NSSI for different purposes, including the desire to alter their internal state; eliminate negative emotions (e.g., anger, stress), negative cognitions (e.g., suicidal ideation, traumatic memories), negative affect states (e.g., dissociation); self-punishment; and to establish a sense of internal control (5, 6, 9, 10).

Although NSSI is generally considered as a behavior associated with psychiatric populations especially borderline personality disorder (1, 11)–several studies have documented it in non-psychiatric samples, including adolescents and young adults (3, 12–14). Furthermore, population-based surveys reported that between 12 and 37.2% of adolescents in secondary school populations and 12 and 20% of teenagers and young adults have engaged in NSSI (15–17). Additionally, the gender differences in NSSI engagement were found to be mixed. While some studies indicate that females frequently engage in NSSI (5, 8), others did not find similar results (1, 7, 11, 18).

Although NSSI has received increased attention in recent years, few studies are concerned with its frequency and functions in different ethnic and cultural groups. The actual prevalence rates of NSSI in community-based populations remain unknown, and the relevant figures tend to vary considerably across studies since operational definitions, and modes of measurement are inconsistent (19–22). Moreover, most studies concerning NSSI are focused on samples from North American and European countries; consequently, little is known about it in other cultures. Only a few studies have focused on non-western cultures and highlighted mental health issues. For example, in Turkey, nearly 20% of high school and 15% of college students reportedly engaged in NSSI (23–25). Similarly, other studies reported that between 22 and 38% of Muslim adolescents and college students engaged in NSSI at some point in their lifetime and that males were more prone to these behaviors (26, 27). In order to fill the gap, this study aimed to examine the prevalence of NSSI among the adolescents in Israel's different ethnic and cultural groups and determine whether these cultural and ethnic backgrounds are related to NSSI.

Several variables act as risk factors for NSSI, including major depression disorder (MDD), anxiety, impulsivity, social isolation, and low self-esteem (5, 11, 28–30). Despite the fact that changes in sleep patterns are one of the most common outcomes of puberty development in comparison to childhood (31) less is known about the relationship between sleep problems and NSSI and whether it is a risk for NSSI. Previous studies suggest these sleep changes can develop sleep problems in many adolescents, which become more frequent with the advancing of puberty (32, 33). Sleep problems are a significant health issue for the youth (33–35), and related to different risk factors that could increase NSSI activities (including depression, anxiety, suicidal ideation, impulsivity, and aggression). Nevertheless, only a few community studies have examined the association between sleep patterns and NSSI. In a population-based study, Hysing et al. (36) found that insomnia, short sleep durations, and an abnormal sleep onset latency were correlated with self injury even after adjusting for demographics, perfectionism, depression, and ADHD symptoms (36). Lundh et al. (37) found poor sleep functions as a risk factor for the development of NSSI among young girls, even when controlling for psychopathology (37). However, their study was not originally designed to investigate the association between sleep and NSSI, and sleep disturbances were measured using only one global question.

Adolescents are especially attracted to technological methods of communication, which offer interaction with others and, at the same time, provide anonymity and a sense of social acceptability (38). The dramatic growth of internet usage among adolescents has increased the prevalence of several related pathologies. For example, internet addictions have become common health issue among adolescents; in the Netherlands and Italy, 3.7 and 5.4% of adolescents exhibit this addiction, respectively (39, 40). Although NSSI and internet addictions are common among adolescents, few studies have described the relationship between these two risk behaviors (41). Nevertheless, significant efforts have been made to understand the relationship between NSSI activity and the contents of materials accessed on the internet (42, 43), including sharing NSSI experiences (44) and communication between people who self-injure in virtual communities (17). Therefore, examining the correlation between the pathological use of the internet and NSSI among adolescents from different ethnicities may provide further knowledge concerning the cross-cultural risk of NSSI.

Taken together, the aims of this study were (1) to explore the prevalence, characteristics, and type of NSSI behavior among adolescents from diverse ethnocultural groups in Israel, and (2) to examine whether sleep problems and internet addictions related to NSSI behaviors when controlling for depressive symptoms.

Our hypotheses were: (1) NSSI will be more frequent among immigrants and adolescents from ethnic minorities than among other population groups. (2) NSSI will be related to sleep difficulties, internet addictions, and depressive symptoms.

## Methods

### Participants and Procedure

This study utilized a cross-sectional design. Furthermore, a sample of 642 adolescents, aged 12–18 years (M = 14.95, SD = 1.53), were recruited from different middle and high schools in Israel by employing a snowball sampling technique (45, 46). The researchers sent a handout to the parents of students, including information regarding the study's aims. Parents were able to update the school administration or the researchers if they did not wish their children to participate in the study. The students whose parents disagreed were excluded. The researchers informed students who chose to participate in the study about the aim of the study, which will take place on a specific day instead of the class scheduled. The adolescents–who chose to participate in the study–were provided with links to local mental health resources. The study was approved by the Ethics Committee of the Ministry of Education of Israel and the IRB of the Academic College of Tel-Aviv-Yaffo.

### Measures

#### Deliberate Self-Harm Inventory-Youth Version

(DSHI-Y) (47). The DSHI-Y is a modified version of the Deliberate Self-harm Inventory (DSHI) (48) that aims to assess the lifetime history of various aspects concerning self injury without suicidal intention. The DSHI is based on the conceptual definition of deliberate self-harm as the deliberate and direct wounding or alteration of body tissue (without conscious suicidal intent) that results in severe injuries which cause tissue damage (48). The DSHI-Y is a 6-item questionnaire that assesses the presence and frequency of the following self-mutilating behaviors: cutting, self-burning, severe scratching, self-biting, banging (of the head and other body parts), and self-punching. Participants rated each item on a 5-point Likert scale, where 1=No, “*I have never done this*;” 2 = “*Yes*, one *time*;” 3 = “*Yes, 2–5 times*;” 4 = “*Yes, 6–10 times*;” and 5 = “*Yes, more than 10 times*.” The questionnaire exhibited excellent internal consistency (α = 0.91). Furthermore, consistent with previous studies, two dichotomous variables were created (37, 47). First, a history of NSSI was established where a score of “0” was assigned for participants who indicated that they had not engaged in any NSSI behaviors and a score of “1” to participants who did have a history of these behaviors. In order to distinguish between frequent and occasional NSSI participants, a second variable was assigned a score of “0” for participants who reportedly engaged in five or fewer NSSI incidents (Infrequent NSSI), and a score of “1” to indicate self-harming participants who reportedly engaged in more than five incidents (frequent NSSI). In the current study, the DSHI-Y's internal consistency was α = 0.78.

#### The Child and Adolescent Sleep Checklist

Oka and Horiuchi (49) is a 24-item checklist designed to identify sleeping habits and screen for sleeping problems in adolescents. Participants were asked to respond to items using the following five choices: 0 indicated “never,” 1 “occasionally,” 2 “sometimes,” 3 “always,” and 4 “unknown.” The global score range was 0–72, where a global score > 18 would indicate the presence of sleeping problems. The reliability of the test ranged between 0.8 and 0.98 (49). In the current study, the CASC's internal consistency was α = 0.81.

#### The Internet Addiction Test

Young (50) is a 20-item questionnaire in which respondents rate items on a five-point Likert scale pertaining to the degree to which their internet usage affects their daily routine, social life, productivity, sleeping pattern, and emotions. Scores range from 20 to 100; Young (50) suggests that a score of 20–39 points typifies an average online user who has complete control over his/her usage; a score of 40–69 signifies frequent problems caused by internet usage, and a score of 70–100 means that their internet usage is causing significant problems. The IAT factor analysis in a previous study revealed a good to moderate internal consistency (α = 0.54–0.82) (51); nevertheless, it has shown a steady internal consistency before (Cronbach's α = 0.93) (52). Consistent with Young's (50) suggestion, dichotomous variables were created in the current study using a 40 or higher cutoff score. A score of “0” and “1” was assigned to participants who reported non-problematic or moderate to severe internet usage, respectively. In this study, the IAT's internal consistency was α = 0.92.

#### Patient Health Questionnaire-9 Modified for Adolescents

Johnson et al. (53) is a 9-item self-report questionnaire to assess the severity of adolescents' depression. Each item is rated on a 4-point scale (0 = Not at all; 1 = Several days; 2 = More than half the days; and 3 = Nearly every day). The total score ranges from 0 to 27; higher scores indicate greater severity of depression. A global score > 10 indicates the presence of moderate to severe depression symptoms. The PHQ-A has shown a 92% accuracy in diagnosing major depression disorder in a past study (53). In the current study, its internal consistency was α = 0.85.

We also assessed participants' demographic characteristics included their age, gender, class, family status, parents' place of birth and educational level, religious affiliation, and degree of religiosity.

### Statistical Analysis

All analyses were conducted using the Statistical Package for the Social Sciences (SPSS) version 20. The comparisons between the NSSI and Non-NSSI groups utilized chi-square tests for dichotomous data (e.g., gender, ethnicity, and other cutoff point measures) and *t*-tests or *ANOVA* for continuous data (sleep problems, the severity of depression, internet addiction). Regression diagnostic tests were used to assess the multicollinearity between predictors. A multiple logistic regression analysis was performed to test the predictive utility of significant correlations for NSSI behaviors. Goodness-of-fit statistics were used to compare and select the most parsimonious models. A Bonferroni correction was administered since the comparison included multiple tests. Furthermore, 90.1% of the participants had no missing data for any variable, 7.2% had missing data for one or two variables, and 3.8 % had missing data for three to four variables. The alpha value was set to 0.05.

## Results

The sample included different ethnic groups: 50% of the participants were Jews (50%; *N* = 321), 34.7% were Muslims (*N* = 223) born in Israel, 9.7% (*n* = 62) were immigrants from the former Soviet Union (FSU), and 4.4% were immigrants from Ethiopia (*N* = 28). The demographic characteristics of the participants are displayed in Table 1.

**Table 1:** Demographic characteristics of the sample (*n* = 642).

**Age M ± SD**	14.95 ± 1.53	
	***N***	**%**
Gender (girls)	344	53.6%
**Ethnicity**	
Jews born in Israel	321	50%
Muslims born in Israel	223	34.7%
FSU immigrants^*^	62	9.7%
Ethiopian immigrants	28	4.4%
**Parents' education level**	
Elementary/ High School Education	267	41.6%
Tertiary academic education	302	47.0%
Level of religiosity (High)	159	24.7%

* *FSU, former Soviet Union*.

**Figure 1:**
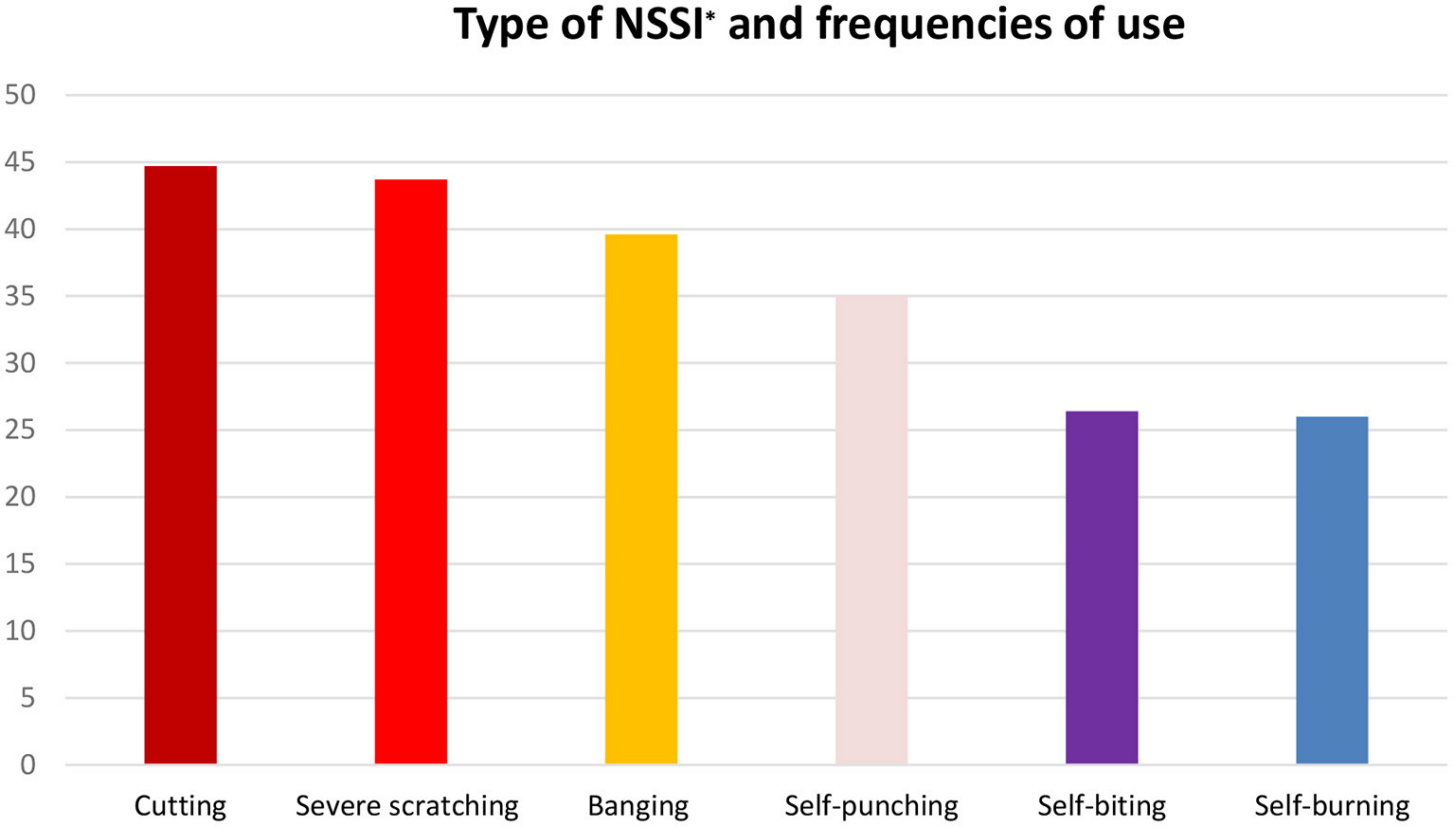
Type of NSSI and frequencies of use. *NSSI, Non-suicidal Self-Injury.

The analyses revealed that 30.7% (*N* = 197) of the participants reported a history of NSSI behavior, while 5.8% (*N* = 37) reported that they frequently engaged in such behavior (i.e., more than five incidences). Furthermore, 26.2% (*N* = 168) of the total sample exhibited severe symptoms of depression, 41.3% (265) reported sleep difficulties, and almost a quarter of them (24.3%) exhibited internet addictions (Table 2). As presented in Figure 1, the most common method of self-mutilation used by the adolescents was cutting (44.7%), followed by severe scratching (43.7% reported severely scratching themselves at least once) and banging (39.6%).

**Table 2:** Clinical characteristics of the participants (*n* = 642).

**Clinical characteristics**		
Depression	168	26.2%
Sleep difficulties	265	41.3%
Internet addiction	156	24.3%
Lifetime NSSI	197	30.7%
Frequent engagement in NSSI	37	5.8%

*NSSI, non-suicidal Self-Injury*.

### Demographic and Clinical Characteristics of Adolescents Engaging in NSSI

The data indicate that adolescents with a history of NSSI behavior were more likely to be male [49.2% (97) vs. 36.9% (162); $\chi_{\left(1\right)}^{2}$ = 8.66, *p* =0.003], FSU immigrants [57.4% (35) vs. 21.9% (70) [or Muslim minority [37.2% (83) vs. 21.9% (70); $\chi_{\left(3\right)}^{2}$ =35.97, *p* < 0.001] when compared to those without such a history. Furthermore, adolescents who engaged with NSSI reported higher levels of depressive symptoms in compare to those who did not engaged in NSSI [42.6% (84) vs. 19.1% (83); $\chi_{\left(1\right)}^{2}$ =38.71; *p* < 0.001], sleep difficulties [59.2% (116) vs. 34.5% (149); $\chi_{\left(1\right)}^{2}$ =33.71; *p* < 0.001], and internet addiction behaviors [37.7% (72) vs. 27% (113); $\chi_{\left(1\right)}^{2}$ =7.15; *p* < 0.01] (Table 3). Within the NSSI group, participants who were frequent engagers (>*5 times*) tended to exhibit higher levels of depressive symptoms [70.3% (26) vs. 36.9% (58); $\chi_{\left(1\right)}^{2}$ =13.55, *p* < 0.001], sleep difficulties [78.4% (29) vs. 55.8% (87); $\chi_{\left(1\right)}^{2}$ =6.37, *P* = 0.01] and more severe internet addictions [45.9% (17) vs. 7.9% (2); $\chi_{\left(1\right)}^{2}$ =14.45, *p* < 0.001].

**Table 3:** Demographic and clinical characteristics of participants' NSSI^*^ history and engagement frequency (*n* = 642).

	**History of NSSI engagement**				**Frequency of NSSI engagement (within NSSI group)**			
	**Without NSSI *N* = 439**	**NSSI** ***N* = 197**	**Test _**(df)**_**	**Significance**	**Low frequency** ***N* = 149**	**High frequency *N* = 37**	**Test _**(df)**_**	**Significance**
**Demographic characteristics**								
Age (M ± SD)	15.0 ± 1.53	14.91 ± 1.51	*t = 0.69*	*p* > 0.1	14.96 ± 1.54	15.14 ± 1.26	*t = −1.01*	*p* > 0.1
Gender (Male) % (*N*)	36.9% (162)	49.2% (97)	$\chi_{\left(1\right)}^{2}$ = 8.66	*P* = 0.003	51% (76)	57.1% (20)	$\chi_{\left(1\right)}^{2}$ = 0.43	*p* > 0.1
Jews born in Israel % (*N*)	78/1% (249)	21.9% (70)^a^	$X_{\left(3\right)}^{2}$ = 35.97	*p* < 0.001	96.2% (306)	3.8% (12)	FET	*p* = 0.08
FSU immigrants^**^ % (*N*)	42.6% (26)	57.4% (35)^b^			89.8% (53)	10.2% (6)		
Ethiopian immigrants% (*N*)	67.95% (19)	32.1% (9)^ab^			96.4% (27)	3.6% (1)		
Muslims born in Israel % (*N*)	62.8% (140)	37.2% (83)^b^			91.9% (205)	8.1% (18)		
Religiosity level (High) % (*N*)	20.3% (89)	25.9% (51)	$\chi_{\left(1\right)}^{2}$ = 3.64	*p* > 0.1	27% (41)	27% (10)	$\chi_{\left(1\right)}^{2}$ = 0.79	*p* > 0.1
**Clinical characteristics % (** ***N*** **)**								
Depression	19.1% (83)	42.6% (84)	$\chi_{\left(1\right)}^{2}$ = 38.71	*p* < 0.001	36.9% (58)	70.3% (26)	$\chi_{\left(1\right)}^{2}$ = 13.55	*p* < 0.001
Sleep problems	34.5% (149)	59.2% (116)	$\chi_{\left(1\right)}^{2}$ = 33.71	*p* < 0.001	55.8% (87)	78.4% (29)	$\chi_{\left(1\right)}^{2}$ = 6.37	*P* = 0.01
Internet addiction	27% (113)	37.7% (72)	$\chi_{\left(1\right)}^{2}$ = 7.15	*p* < 0.01	7.9% (12)	45.9% (17)	$\chi_{\left(1\right)}^{2}$ = 14.45	*p* < 0.001

* *NSSI, Non-suicidal Self-Injury*.

** *FSU, former Soviet Union; FET, fisher's exact test*.

A multiple logistic regression analysis was performed to test the predictive utility of the significant correlates concerning engaging in NSSI. As it can be seen on Table 4, when controlling for the above-noted demographic and depressive symptoms, the most parsimonious model set included the following variables: being male (OR = 2.39, *P* < 0.001), immigrants (OR = 2.15, *p* < 0.001), Muslims born in Israel who also exhibit severe depressive symptoms (OR = 2.34, *p* < 0.001), and people with internet addictions (OR = 1.01, *p* = 0.005).

**Table 4:** Logistic regression model predicting NSSI (*n* = 642).

	**Odds ratio**	**S.E**.	**Wald z**	***P*-value**	**95% CI**
FSU immigrants^*^	2.15	0.19	14.78	<0.001	1.45–3.18
Muslims born in Israel^*^	1.78	0.24	12.54	0.002	0.92–2.86
Gender (Male)	2.39	0.20	18.53	<0.001	1.61–3.56
Depression	2.34	0.23	14.11	<0.001	1.50–3.64
Internet addiction	1.01	0.06	7.59	0.005	1.0–1.03

* *Reference group, Jewish adolescents born in Israel. Hosmerand Lemeshow test for goodness-of-fit: $\chi_{\left(8\right)}^{2}$ = 9.64, p = 0.26*.

## Discussion

This study is one of the few studies focusing on a non-clinical adolescent sample from a diverse ethnic adolescent community and aims to identify its associated risk factors. The results revealed that almost one-third of the participants engaged in NSSI, while 6% frequently injured themselves. More than half of the FSU immigrants and over one-third of the Muslim participants reportedly engaged in NSSI. Furthermore, adolescents from ethnocultural minority backgrounds (FSU immigrants and Muslims born in Israel) exhibited severe depressive symptoms and internet addictions.

The prevalence of NSSI among the sample participants exhibited relatively higher rates when compared to both Western and non-Western samples. For instance, the lifetime frequency of NSSI among adolescents in western countries ranges between 13.9 and 35.6% (1, 3, 7, 54). The same trend was found in non-Western countries as well, where the estimated lifetime frequencies varied from 9.3% in Japan (55) to 32.7% in Hong Kong (56).

Furthermore, the results indicate that immigrants and Muslim minority adolescents had a higher risk of engaging in NSSI than Israeli Jewish adolescents. These results are significant since they indicate inconsistent NSSI prevalence results between ethnic and racial groups. Interestingly, while some studies did not find any differences (57, 58), others found higher rates of NSSI among white participants when compared to ethnic minority individuals (17, 59–61). Only one other study reported higher frequencies of NSSI among minority groups (62). The first and second generations of FSU immigrants and immigrants from Ethiopia were more at risk of suicidal behaviors (63), alcoholism, and substance abuse than their native counterparts (64). Different models may explain why these groups are vulnerable to psychological distress and risk behaviors. One possible explanation is related to “*minority stress*,” which refers to stress experiences during adverse social interactions resulting from being a stigmatized social group and the target of discrimination and prejudice (65). This may affect their vulnerability toward adverse psychological, social, and academic outcomes; this includes suicidal behavior, depression, anxiety, and delinquency among immigrants and ethnic minority groups (66–71).

In our study, the Muslim adolescents–who live as an ethnic minority in Israel–were more vulnerable to psychopathology and risk behaviors, including depression, and somatization, than the Jewish students, as reported in different studies (67). Furthermore, Muslim adolescents were found significantly more at risk for suicidal behaviors than their counterparts (72). One explanation for these patterns is related to the fact that they may be exposed to more stressors and the lack of mental health resources. Another explanation could be related to the infrequent help-seeking behaviors of immigrants and ethnic minority adolescents. Several studies assert that ethnicity and gender are significant determinants of help-seeking (73–76). Accordingly, the fact that the current study participants who engaged in NSSI were more likely to be male and ethnic and cultural minorities minimized their probability of seeking help. Different hypotheses have been suggested regarding the barriers against help-seeking among adolescents and young men. These include denial of emotions (77, 78), avoidance (79), and perceived stigma (80). Therefore, a future study that focuses on help-seeking patterns and attitudes toward mental health difficulties of male adolescents from different ethnic and cultural backgrounds may clarify the roles of gender, culture and norms in preventing and treating psychopathology.

The results of this study highlight that adolescents who injure themselves suffer from sleep difficulties and internet addictions. It can be suggested that the high correlation between the participants' internet addictions and sleep problems negatively affects their ability to regulate emotions (81) and increases their depressive symptoms. There is evidence, albeit not definitive, that interfering with sleep is a causal pathway between excessive social media use and NSSI (82). Therefore, it is imperative to determine how different aspects of social media use (duration and timing) are related to sleep problems, psychopathology, and suicidal risk. Sleep hygiene programs implemented in high schools were found to help raise sleep efficacy, reduce emotional distress and risk behaviors, and sustain more stable academic performance than those who did not participate in the programs (83).

Furthermore, vulnerable youths who injure themselves tend to struggle with naming their emotions, coping with difficulties and are more likely to seek cyber support and information than professional help (84). However, the virtual communities they are attracted to could increase their risk of normalizing or even encouraging risk behaviors, including NSSI (85). Despite these dangers, the relationship between social media and NSSI remains unclear.

The current study has several significant limitations. First, in using a cross-sectional survey, causality among the study variables should not be inferred. More specifically, the temporal effect of internet usage on depression and NSSI could not be determined. Second, all study measures were self-reported and susceptible to subjectivity biases and under reporting due to social desirability. Third, the study's sample comprised adolescents' volunteers, perhaps not accurately representing the adolescents' population in Israel. This is especially true regarding the immigrants from Ethiopia with a low percentage in our sample. Future studies are needed to examine the research questions in longitudinal studies with at least some objective measures.

This study's results emphasize the need to routinely assess NSSI, especially among immigrants and ethnocultural minority groups and in non-clinical samples. An earlier age of onset of NSSI and a longer duration of NSSI during adolescence predict adult borderline personality disorder (BPD) (86). Furthermore, different studies have presented that engaging NSSI behaviors among adolescents are a dominant and unique risk factor for all forms of suicidal thoughts and behaviors (87, 88). Therefore, primary preventive programs that include information concerning adaptive coping skills may eliminate its social contagion effect (89). Furthermore, cultural adaptation should be geared toward improving the validity of these programs. Lastly, the study highlights the importance of sleep hygiene and preventive programs that reduce the adverse consequences of sleep interference.

## Data Availability Statement

The raw data supporting the conclusions of this article will be made available by the authors, without undue reservation.

## Ethics Statement

The studies involving human participants were reviewed and approved by Academic College of Tel Aviv-Yaffo. Written informed consent to participate in this study was provided by the participants' legal guardian/next of kin.

## Author Contributions

All authors listed have made a substantial, direct, and intellectual contribution to the work and approved it for publication.

## Conflict of Interest

The authors declare that the research was conducted in the absence of any commercial or financial relationships that could be construed as a potential conflict of interest.

## Publisher's Note

All claims expressed in this article are solely those of the authors and do not necessarily represent those of their affiliated organizations, or those of the publisher, the editors and the reviewers. Any product that may be evaluated in this article, or claim that may be made by its manufacturer, is not guaranteed or endorsed by the publisher.
